# Association of Antepartum Perceived Maternal Social Support with Postpartum Depression among women in Mbarara district, rural southwestern Uganda

**DOI:** 10.21203/rs.3.rs-7020757/v1

**Published:** 2025-08-07

**Authors:** Catherine Atuhaire, Kabanda Taseera, Daniel Atwine, Samuel Maling, Vikram Patel

**Affiliations:** Mbarara University of Science and Technology; Mbarara University of Science and Technology; SRF Research and Training Centres; Mbarara University of Science and Technology; Harvard Medical School

**Keywords:** Postpartum depression, maternal social support, pregnancy, rural Uganda, mental health, perinatal care

## Abstract

**Background:**

Postpartum depression (PPD) remains a global maternal mental health concern, particularly in low-income settings. Maternal social support has been identified as a potential protective factor, yet little prospective evidence exists from rural Uganda. This study examined the association between Antepartum Perceived Maternal Social Support and Postpartum Depression among women in rural southwestern Uganda.

**Methods:**

A multi-facility prospective cohort study was conducted among pregnant women attending three health facilities in Mbarara District, rural Southwestern Uganda. Participants were enrolled during latent labor and followed up at six weeks postpartum. Perceived Maternal Social Support was measured using the Maternity Social Support Scale (MSSS), and PPD was assessed using the Edinburgh Postnatal Depression Scale (EPDS) and the MINI International Neuropsychiatric Interview. These were assessed antepartum in latent labour and 6 weeks following childbirth. Data were analyzed using logistic regression in both univariable and multivariable analyses.

**Results:**

A total of 502 pregnant women were included in this analysis aimed at establishing the association between Antepartum Perceived Maternal Social Support with Postpartum Depression. The overall prevalence of PPD was 4.8%. Inadequate social support was reported by 46% of participants. Bivariate analysis showed a great association between inadequate social support and PPD (OR: 0.3; 95% CI: 0.1–0.8; p = 0.017). In multivariate analysis, adequate maternal social support was associated with a 60% reduction in the odds of developing postpartum depression (OR: 0.4; 95% CI: 0.2–1.0; p = 0.056). Other significant predictors of postpartum depression were experiencing complications during delivery, preterm birth, and stressful life events during labour.

**Conclusion:**

Perceived Maternal Social Support is a protective factor against Postpartum Depression. Strengthening social support systems during the antepartum period and the early postpartum period may help reduce the burden of PPD among women, especially those living in rural settings where access to mental health resources may be limited.

## BACKGROUND

Postpartum depression (PPD) has remained a global maternal mental health concern and a contributing factor to maternal morbidity with severe negative impact on the mother, the newborns, family and community at large(Wang, 2021). It accounts for 10–15% population Globally and a higher prevalence has been documented in low-income countries (LICs) (Wang, Z et al,2021). For instance, it has been reported at 27% in southwestern Uganda (Atuhaire, 2021).

The actual cause of PPD among Ugandan women is not clearly known, but an association of PPD with lack of social support was recently reported in a cross sectional study in south western Uganda (6)(7). Social support is the assistance, care, and resources provided by individuals or groups to help mothers navigate the physical, emotional, and psychological challenges during the postpartum period. This support plays a critical role in promoting maternal health and its attributed to reducing the risk of PPD (Tambağ, 2018). Social support to postpartum mothers is got from the spouse, family members and friends, it is in form of moral support, giving a hand in care and counselling. In some studies, Perceived Maternal Social Support (PMSS) depends on who provides it; the partner, mother, in-laws or friends and type of social support received like helping with house chores, babysitting, financial support (Özmen et al., 2014), however, these studies have been carried out in high-income countries.

The postpartum care package includes care within 48 hours, at seven days and at six weeks following childbirth (Kikuchi et al., 2015). This indicates that to achieve the Sustainable Development Goal (SDG) number three in which maternal mortality ratio (MMR) is targeted to; reduce to less than 70 deaths/100,000 live births (Callister & Edwards, 2017), reduce the neonatal mortality rate (NMR) to less than 7/1000 live births by the year 2030, there must be robust actions taken at all stages of perinatal care inclusive of postpartum for improved quality and uptake of the services.

Despite the government initiative to address the contributing factors of PPD by improving the financial status of mothers through parish development models, gender equality and women empowerment, PPD has remained high in south western Uganda. Besides perceived social support, a preliminary cross-sectional study suggested that other factors like mothers who are Human Immunodeficiency Virus (HIV) positive, rural residence of the mother, complication in recent pregnancy and baby crying excessively were associated with PPD.

Of the associated factors identified above, social support is a modifiable factor where an intervention can be done to improve PPD. Given that prior studies were cross sectional in nature, they were unable to examine the temporal causal relationship. We therefore investigated the level of Perceived Maternal Social Support and its subsequent association with Postpartum Depression within 6 weeks following delivery.

## METHODS

### Study design and setting:

We conducted a multi-facility prospective cohort from November 2023 and March 2024. This study was conducted in three health facilities in Mbarara district, rural southwestern Uganda: Mbarara Regional Referral Hospital (MRRH), Bwizibwera Health Center IV (BHC IV), and Mbarara Municipal Health Center IV (MMHC IV).

MRRH is government owned referral hospital and a teaching hospital for the Medical and Nursing School of Mbarara University of Science and Technology. The hospital was founded in 1940 and it has a bed capacity of 600 and their annual enrollment is 17400 patients, while the outpatients seen are 63300. The Mbarara city is situated approximately 270 kilometers south west of Kampala, the capital city of Uganda. Currently, the hospital serves a population of over four million people in its catchment area. The catchment population for MRRH is from 12 districts including Mbarara district of the south western region. Every day, the hospital sees about 1,200–1,500 patients. There are 3 nurses on antenatal clinic and an average monthly attendance of 400 pregnant women (MRRH records).

BHC IV is a health center that is based in a sub-urban region but serves the rural community in Kashari county, Mbarara district. It has eight midwives and an average daily attendance of 15 mothers. Approximately 300 pregnant women visited BHC IV in the previous six months (health facility records).

MMHC IV is located in Mbarara city, Mbarara district. It has 3 nursing staff on antenatal clinic and an average monthly attendance of 200 pregnant women (health facility records).

### Study population

We targeted to enroll pregnant women in latent labor attending any of the 3 recruitment sites, that provided a written informed consent and residing within a radius of 20km from the targeted health facilities. This limit on distance allowed the study investigators to be able to follow up these mothers in their communities. We excluded mothers who were in active labor and those on treatment for depression.

### Sample size estimation:

We calculated a sample size of 506 pregnant women based on the sample size calculation for establishing a single population parameter (Kelsey, J.L. et al).

We estimated a sample size of 506 pregnant women in latent labor using the formula for estimation of single population proportion, n = Z_α/2_^2^ *P (1 - P)/r^2^ (Kelsey, J.L. et al) to estimate a prevalence of PPD of 27.1% (Atuhaire, 2021) with assumptions of Z_α/2_ of 1.96, margin error (r ) of estimation of 5% (0.05) and a predicted 10% loss to follow-up and missing data and considering a design effect of 1.5 to cater for non-random sampling (consecutive).

#### Sampling strategy

Using proportionate stratified consecutive sampling method, the sample size was divided proportionately among the study sites with the site as a stratum. This was based on the number of pregnant women visiting the sites in the previous months as declared by the facility records. We estimated the proportion of pregnant women in each stratum and using probability proportionate to size, we determined the number of mothers to enroll at each site in order to meet the sample size required. Women within each site were selected consecutively based on eligibility criteria until the sample size apportioned to each stratum was attained.

#### Data collection

At baseline, all pregnant women in latent labour who meet the eligibility criteria were consented and enrolled after they had been admitted for routine care on maternity wards of the health facilities. These women were identified by the research team of graduate nurses placed in each health facility and directed to a private room or in an open private space within the compound for consenting and data collection. They underwent a face-to-face interview using a pretested questionnaire to collect their sociodemographic, medical, obstetric, psychosocial and Newborn characteristics. The interviews were conducted by trained research assistants using English or runyankore (local language) based on the participant’s preference.

They were all screened and assessed for depression and perceived maternal social support. This assessment was repeated at 6 hours, Day 6 and 6th week postpartum. On day 6, those who were still admitted in the hospital, were assessed for depression and perceived social support. Women who were discharged to their homes, were followed up on a phone call and assessed for the same. These women were still followed up at 6 weeks where the study team assessed for PPD and perceived maternal social support during the postnatal clinic as mothers brought their children for immunization.

### Measurements:

#### Primary Predictor:

Perceived Maternal Social Support was measured at baseline during latent labor, 6th hour, Day 6 and at 6th week postpartum using the Maternity Social Support Scale (MSSS) (Webster *et al.*, 2000). This tool measures the extent to which these mothers receive perceived social support from family, partners and friends and it was validated to be used in this setting (Dibaba, Fantahun and Hindin, 2013). MSSS is a brief, easy-to-administer self-report questionnaire consisting of six items scored on a 5-point frequency Likert scale of which 1 is never and 5 is always. Scoring for two items is reversed to minimize bias and careless answering of the items. Scores range from 0 to 30 with scores categorized as low (0–18), medium (19–24) and adequate (> 24). For this study, it has been categorized as inadequate (0–24) and adequate (> 24) with higher scores denoting adequate perceived social support (Webster *et al.*, 2000). The exposed group were mothers with adequate perceived MSS and the unexposed group, inadequate perceived MSS. This tool measures three types of social support; emotional/informational support, tangible support and affectionate support (Webster *et al.*, 2000). It has internal consistency of Cronbach’s alpha value documented as between 0.71 and 0.90 (Mohammad *et al.*, 2018).Primary Outcome: Postpartum depression at the 6th week using the validated EPDS and the Mini International Psychiatric Interview (M.I.N.I 7.0.2) (Sheehan *et al.*, 1994). This interview is a “gold standard” that determines objective clinical diagnoses of PPD according to the Diagnostic and Statistical Manual of Mental Disorders 5th edition classification (DSM-5) (First, Williams, Karg, & Spitzer, 2015). It was previously validated and used among postpartum mothers in Mbarara district and other low-income settings (Haque, Namavar and Breene, 2015; Gebregziabher et al., 2020; Okunola et al., 2022) and scores are a binary outcome with “Yes” or “No” postpartum depression.

### Quality assurance:

To ensure data quality, the Principal Investigator (PI) closely supervised the data collection process to ensure adherence to the sampling procedure and proper administration of the tools. The study team reviewed completed questionnaires immediately after the interviews to verify completeness and accuracy. All study tools were validated prior to use.

The PI developed a Standard Operating Procedures (SOPs) manual to guide the entire study process. She, along with the research assistants, was actively involved from the beginning to the end of the study at all three sites. Additionally, the PI ensured that all research assistants received comprehensive training on study procedures, including participant screening, recruitment, and obtaining informed consent.

### Data analysis:

Data was entered into a database designed with KOBO Collect software, cleaned and verified. The dataset was imported into STATA© 14.0 software (College Station, Texas, USA) for analysis. Participants’ characteristics were described using means or medians for continuous variables and proportions for categorical variables and presented in a table.

To examine the relationship between perceived maternal social support and postpartum depression, we employed logistic regression in both univariable and multivariable analyses. The measures of association were reported as adjusted and unadjusted odds ratios with their corresponding 95% confidence intervals. To account for potential confounding variables, we included such factors as control variables in our analysis. All analyses were performed using STATA version 14.

## RESULTS

A total of 502 pregnant women were included in this analysis aimed at establishing the association between antepartum maternal social support with postpartum depression. Of these, 224 (44.6%), 165 (32.9%), and 113 (22.5%) were enrolled from Mbarara Regional Referral Hospital, Bwizibwera Health Centre IV and Mbarara Municipal Health Centre IV respectively. (Table 1)

### Participants’ characteristics

The participants had a mean age of 26.1±5.4 years with majority aged 15-29 years and in marital unions (95.4%). Slightly more than half of participants had attained at least a secondary level of education (52.8%), unemployed (52.6%), and with no or less than 100,000 Ugandan shillings as their monthly income (Table 1).

### Mother’s obstetric, psychosocial and Newborn characteristics

About two-thirds of mothers had spontaneous vaginal delivery (61.8%). Majority reported to having been happy about having delivered (98.8%). Majority of male partners felt happy with current delivery (96.2%). Majority of newborns were term-babies (98%), had a male to female ratio of 1:1, with majority having a 10-minute Apgar score of 7-10 (95.4%) and breastfeeding well (96%) (Table 1).

### Overall prevalence of Postpartum Depression and Perceived Maternal Social Support score among mothers in Mbarara

Of the 502 women enrolled and included in this analysis, 24 were diagnosed with postpartum depression giving a prevalence of 4.8% (95% CI:3.2-7.0) with no significant age disparities (p>0.05). Inadequate maternal social support (MSS) was noted in 231 mothers giving a prevalence of 46% (95% CI: 41.7 – 50.4) with no age-specific disparities (0.094) Table 2.

### Association between Antepartum Perceived Maternal Social Support with Postpartum Depression

In bivariate analysis, Perceived Maternal Social Support within 6 weeks’ postpartum was significantly associated with Postpartum Depression at 6 weeks among mothers (p=0.017). Other factors that showed a statistically significant association with PPD are; Mother having had complications during the delivery (p=0.012), Baby experiencing any health problems since birth (p=0.042), Had their baby at <9months (p=0.009), Had baby hospitalized in the last 6 days (p=0.034), Breastfeeding well (p=0.042), Baby difficult to console or sooth (p=0.029), Happy with the baby (p=0.006), and any Stressful life events during labour (p<0.001) (Table 1).

In multivariate analysis, Perceived Maternal Social Support within 6 weeks’ postpartum was associated with a 60% reduction in odds of having PPD (OR=0.4; 95% CI: 0.2 – 1.0) with a tendency towards significance (p=0.056) after adjusting for other factors in the model.

Other factors with an independent association with PPD at 6 weeks’ among mothers were; mother having had complications during the delivery, having had their baby at <9months and having had any stressful life events during labour.

Mothers reporting to have had complications during the delivery had 4.3 times higher odds of having PPD at 6 weeks (OR=4.3; 95% CI: 1.1 – 16.8) as compared to those without, p=0.037. Mothers reporting to have had their baby at 9 months had 90% lower odds of having PPD at 6 weeks (OR=0.1; 95% CI: 0.0 – 0.6) as compared to those without, p=0.011. Mothers reporting to have had any stressful life events during labour had 11.4 times higher odds of having PPD at 6 weeks (OR=11.4; 95% CI: 3.3 – 39.1) as compared to those without, p<0.001 (Table 3).

## DISCUSSION

This study examined the association between Antepartum Perceived Maternal Social Support and Postpartum Depression among women in rural southwestern Uganda. The null hypothesis stated that PMSS during and after pregnancy is not associated with the development of PPD. However, the findings of this study specify that inadequate Perceived Maternal Social Support is significantly associated with a higher risk of developing PPD. This aligns with previous studies suggesting that social support plays a crucial role in maternal mental health outcomes (Tambag, Turan, Tolun, & Can, 2018). Other factors with an independent association with PPD at 6 weeks’ among mothers were; mother having had complications during the delivery, having had their baby at < 9months and having had any stressful life events during labour.

The prevalence of PPD in this study was 4.8%, lower than the 27.1% previously reported in southwestern Uganda. “This did not vary by the number of previous births or mode of birth. Five factors associated with PPD were low perceived social support, HIV positive status, rural residence, obstetrical complications, and the baby crying excessively (Atuhaire et al., 2021). The discrepancy in this study may be due to differences in the study design (this being a longitudinal study vs the cross sectional in the previous study) and population characteristics. This study employed the validated Edinburgh Postnatal Depression Scale (EPDS) and Mini International Psychiatric Interview (M.I.N.I 7.0.2), which have been widely used in similar settings contributing to reliability of our findings (Gebregziabher et al., 2020; Okunola et al., 2022). Additionally, improved maternal mental health awareness and interventions in the study region may have contributed to the lower prevalence.

In bivariate analysis, inadequate PMSS was greatly associated with PPD (OR: 0.3; 95% CI: 0.1–0.8; p = 0.017). After adjusting for potential confounding factors in multivariate analysis, women with adequate Perceived Maternal Social Support still demonstrated a 60% reduced odds of developing PPD (OR: 0.4; 95% CI: 0.2–1.0), with borderline statistical significance (p = 0.056). This finding supports existing literature that highlights the protective role of social support in maternal mental health. (Webster, Nicholas, Velacott, Cridland, & Fawcett, 2011) highlights that women with low social support are more likely to report Postpartum Depression and lower quality of life than well-supported women. In a study by (Mohd, Yunus, Hairi, Hairi, & Choo, 2019), findings emphasized the association between good social support and decreased depression among older adults. The different types of support, including emotional, informational, tangible, and affectionate support have been shown to reduce stress and enhance coping mechanisms, lowering the risk of PPD (Dibaba, Fantahun, Hindin, & childbirth, 2013; Ozmen, Cetinkaya, Ulas, Ozmen, & Research, 2014).

Complications during childbirth was another significant factor independently associated with PPD. Women who experienced complications had over four times the odds of developing PPD compared to those with uneventful deliveries. This is consistent with prior studies that reported childbirth complications like emergency caesarian section, prolonged labour and pre-eclampsia as major risk factor for postpartum mental health disorders (Kim, Park, & Nho, 2022). The heightened physical and emotional distress associated with complicated deliveries is likely to contributes to increased vulnerability to PPD. This is because these complications may intensify physical and emotional stress, disrupt the birth experience and may signal ongoing health or parenting challenges.

In addition, having a term baby was found to be strongly protective against PPD and this may be due to reduced neonatal complications and decreased maternal anxiety. Previous studies suggest that preterm birth and neonatal complications can heighten maternal stress and anxiety, which in turn increases the likelihood of experiencing depressive symptoms (Ntaouti et al., 2020). This maybe because it involves medical emergencies that alerts mothers how vulnerable their infants are, leads to emotional trauma and bonding difficulties hence demanding intensive care giving and lifestyle adjustments. These findings highlight the importance of prenatal care and interventions that support mothers at risk of preterm delivery.

Stressful life events during labor emerged as the most potent predictor of PPD. This finding supports previous research that emphasizes the impact of acute psychosocial stressors on maternal mental health issues and the potential for long-term emotional consequences if unaddressed (Rezaie-Keikhaie et al., 2020). The postpartum period is already a sensitive time with significant physical recovery, hormonal shifts, and identity changes of a mother. Adding stressful life events like having no job, financial constraints, death of a loved one or domestic violence is likely to promote development of PPD. Moreover, these stressful events may exhaust personal coping mechanisms thereby triggering symptoms of depression. In a nutshell, it is explained by the fact that stressful life events may intensify emotional and physical stress during this vulnerable postpartum period, it may reduce coping capacity, trigger biological responses linked to mood disorders and may interfere with self-perception of incapability as a care giver to the newborn. Addressing psychosocial stressors through mental health counseling and support networks may help reduce the incidence of those at risk of developing PPD.

### Implications

These findings underscore the importance of integrating maternal social support interventions into routine maternal care across the lifespan of peripartum. Doctors, midwives, nurses, and community health workers (village health teams) should be trained to assess social support levels and identify mothers at risk of postpartum depression as early as possible.

Community and family-based support systems, including male partner involvement, should be promoted to strengthen maternal mental health.

Additionally, early detection of complications and stressful life events during labour and tailored psychosocial counselling may reduce the risk of postpartum depression and improve maternal and newborn outcomes.

## Conclusion

Perceived Maternal Social Support within 6 weeks’ postpartum is a protective factor against Postpartum Depression. Inadequate maternal social support (MSS) was noted in 231 mothers giving a prevalence of 46%. A mother having had complications during the delivery, a mother having had their baby at < 9months and a mother having had any stressful life events during labour were other factors that are significantly associated with PPD at 6 weeks.

## Figures and Tables

**Table 1:** Bivariate analysis of sociodemographic, obstetric, psychosocial, and newborn factors associated with antepartum maternal social support and postpartum depression at 6 weeks among mothers in Mbarara district

Variable	Overall n (%)	Inadequate MSS n (%)	Adequate MSS n (%)	UOR (95%CI)	p value
MSS Score within 6 weeks’ postpartum					
Inadequate	231 (46.1)	214 (92.6)	17 (7.4)	1.0	
Adequate	271 (53.9)	264 (97.4)	7 (2.6)	0.3 (0.1 – 0.8)	**0.017**

Education level					
Primary or no education	237 (47.2)				
Secondary	212 (42.2)				
Tertiary	53 (10.6)				

Marital Status					
Unmarried	23 (4.6)				
Married	479 (95.4)				

Occupation					
Unemployed	264 (52.6)				
Business	139 (27.7)				
Peasant Farmer	78 (15.5)				
Professional	21 (4.2)				

Monthly income					
None	68 (13.6)				
<100,000UgShs	230 (45.8)				
>100,000UgShs	204 (40.6)				

Age category in years					
15 - 29	378 (75.3)	359 (95.0)	19 (5.0)	1.0	
30 – 49	124 (24.7)	119 (96.0)	5 (4.0)	0.8 (0.3 – 2.2)	0.653

Mode of delivery					
SVD	310 (61.8)	297 (95.8)	13 (4.2)	1.0	
Caesarian Section	192 (38.2)	181 (94.3)	11 (5.7)	1.4 (0.6 – 3.2)	0.435

Mother had complications during the delivery					
No	482 (96.1)	462 (95.9)	20 (4.1)	1.0	
Yes	20 (3.9)	16 (80.0)	4 (20.0)	5.8 (1.8 – 18.9)	**0.012**

Sex of the Baby					
Male	251 (50.0)	241 (96.0)	10 (4.0)	1.0	
Female	251 (50.0)	237 (94.4)	14 (5.6)	1.4 (0.6 – 3.3)	0.405

Apgar score of the baby					
7-10	479 (95.4)	458 (95.6)	21 (4.4)	1.0	
4-6	22 (4.4)	19 (86.4)	3 (13.6)	3.4 (0.9 – 12.6)	0.061
Below 4	1 (0.2)	1 9100.0)	0 (0.0)	1.0 (NA)	

Any newborn abnormalities or complications at birth					
No	476 (94.8)	455 (95.6)	21 (4.4)	1.0	
Yes	26 (5.2)	23 (88.5)	3 (11.5)	2.8 (0.8 – 10.2)	0.121

Baby experiencing any health problems since birth					
No	482 (96.0)	461 (95.6)	21 (4.4)	1.0	
Yes	20 (4.0)	17 (85.0)	3 (15.0)	3.9 (1.1 – 14.3)	**0.042**

Had your baby at 9months (term baby)					
No	10 (2.0)	7 (70.0)	3 (30.0)	0.1 (0.0 – 0.4)	**0.009**
Yes	492 (98.0)	471 (95.7)	21 (4.3)	1.0	

Had baby hospitalized in the last 6 days					
No	483 (96.2)	462 (95.7)	21 (4.3)	1.0	
Yes	19 (3.8)	16 (84.2)	3 (15.8)	4.1 (1.1 – 15.3)	**0.034**

Baby Breastfeeding well					
No	20 (4.0)	17 (85.0)	3 (15.0)	3.9 (1.1 – 14.3)	**0.042**
Yes	482 (96.0)	461 (95.6)	21 (4.4)	1.0	

Baby difficult to console or sooth					
No	493 (98.2)	471 (95.5)	22 (4.5)	1.0	
Yes	9 (1.8)	7 (77.8)	2 (22.2)	6.1 (1.2 – 31.2)	**0.029**

Partner’s feelings on current delivery					
Not happy	19 (3.8)	17 (89.5)	2 (10.5)	1.0	
Happy	483 (96.2)	461 (95.5)	22 (4.5)	0.4 (0.1 – 1.8)	0.229

Mother Happy with the baby					
Very Happy	280 (55.8)	264 (94.3)	16 (5.7)	2.1 (0.8 – 5.6)	
Happy	218 (43.4)	212 (97.3)	6 (2.7)	1.0	
A little unhappy	2 (0.4)	1 (50.0)	1 (50.0)	35.3 (2.0–634.6)	**0.006**
Very unhappy	2 (0.4)	1 (50.0)	1 (50.0)	35.3 (2.0–634.6)	

Any Stressful life events during labour					
No	487 (97.0)	469 (96.3)	18 (3.7)	1.0	
Yes	15 (3.0)	9 (60.0)	6 (40.0)	17.4 (5.6 – 54.1)	**<0.001**

NA: Not available; OR, Odds Ratio; UOR, Unadjusted Odds Ratio; MSS, Maternal Social Support; CI, Confidence interval. SVD, Spontaneous Vaginal Delivery.

**Table 2:** Prevalence of Postpartum Depression at 6 weeks postpartum and Perceived Maternal Social Support in Mbarara district

Prevalence	N	n	% (95%CI)	p value
**Postpartum depression**				
Overall prevalence	502	24	**4.8** (3.2 – 7.0)	

Maternal age category in years	378			0.653
15 - 29		19	5.0 (3.2 – 7.8)	
30 – 49		5	4.0 (1.7 – 9.4)	

**Perceived Maternal Social Support Score**				
MSS Score within 6 weeks’ postpartum	502			
Inadequate		231	**46.0** (41.7 – 50.4)	
Adequate		271	54.0 (49.6 – 58.3)	

Maternal age category in years	378			0.094
15 - 29		196	51.9 (46.8 – 56.9)	
30 – 49		75	60.5 (51.5 – 68.8)	

**Table 3:** Showing the Multivariate analysis associated with Perceived Maternal Social Support and Postpartum Depression at 6 weeks among mothers in Mbarara district

Variable	Adjusted OR (95% CI)	p value
MSS Score at 6 weeks		
Inadequate	1.0	
Adequate	0.4 (0.2 – 1.0)	0.056

Had complications during the delivery		
No	1.0	
Yes	4.3 (1.1 – 16.8)	0.037

Had your baby at <9months		
No	1.0	
Yes	0.1 (0.0 – 0.6)	0.011

Any Stressful life events during labour		
No	1.0	
Yes	11.4 (3.3 – 39.1)	<0.001

## Data Availability

All relevant data are included in the manuscript and all the data underlying the study findings will be available without restrictions if requested from the first and corresponding author, Catherine Atuhaire.
